# A mitochondria-targeted fatty acid analogue influences hepatic glucose metabolism and reduces the plasma insulin/glucose ratio in male Wistar rats

**DOI:** 10.1371/journal.pone.0222558

**Published:** 2019-09-24

**Authors:** Carine Lindquist, Bodil Bjørndal, Hege G. Bakke, Grete Slettom, Marie Karoliussen, Arild C. Rustan, G. Hege Thoresen, Jon Skorve, Ottar K. Nygård, Rolf Kristian Berge

**Affiliations:** 1 Department of Clinical Science, University of Bergen, Bergen, Norway; 2 Department of Heart Disease, Haukeland University Hospital, Bergen, Norway; 3 Section for Pharmacology and Pharmaceutical Biosciences, Department of Pharmacy, University of Oslo, Oslo, Norway; 4 Department of Pharmacology, Institute of Clinical Medicine, University of Oslo, Oslo, Norway; Consiglio Nazionale delle Ricerche, ITALY

## Abstract

A fatty acid analogue, 2-(tridec-12-yn-1-ylthio)acetic acid (1-triple TTA), was previously shown to have hypolipidemic effects in rats by targeting mitochondrial activity predominantly in liver. This study aimed to determine if 1-triple TTA could influence carbohydrate metabolism. Male Wistar rats were treated for three weeks with oral supplementation of 100 mg/kg body weight 1-triple TTA. Blood glucose and insulin levels, and liver carbohydrate metabolism gene expression and enzyme activities were determined. In addition, human myotubes and Huh7 liver cells were treated with 1-triple TTA, and glucose and fatty acid oxidation were determined. The level of plasma insulin was significantly reduced in 1-triple TTA-treated rats, resulting in a 32% reduction in the insulin/glucose ratio. The hepatic glucose and glycogen levels were lowered by 22% and 49%, respectively, compared to control. This was accompanied by lower hepatic gene expression of phosphenolpyruvate carboxykinase, the rate-limiting enzyme in gluconeogenesis, and *Hnf4A*, a regulator of gluconeogenesis. Gene expression of pyruvate kinase, catalysing the final step of glycolysis, was also reduced by 1-triple TTA. In addition, pyruvate dehydrogenase activity was reduced, accompanied by 10-15-fold increased gene expression of its regulator pyruvate dehydrogenase kinase 4 compared to control, suggesting reduced entry of pyruvate into the TCA cycle. Indeed, the NADPH-generating enzyme malic enzyme 1 (ME1) catalysing production of pyruvate from malate, was increased 13-fold at the gene expression level. Despite the decreased glycogen level, genes involved in glycogen synthesis were not affected in livers of 1-triple TTA treated rats. In contrast, the pentose phosphate pathway seemed to be increased as the hepatic gene expression of glucose-6-phosphate dehydrogenase (G6PD) was higher in 1-triple TTA treated rats compared to controls. In human Huh7 liver cells, but not in myotubes, 1-triple-TTA reduced glucose oxidation and induced fatty acid oxidation, in line with previous observations of increased hepatic mitochondrial palmitoyl-CoA oxidation in rats. Importantly, this work recognizes the liver as an important organ in glucose homeostasis. The mitochondrially targeted fatty acid analogue 1-triple TTA seemed to lower hepatic glucose and glycogen levels by inhibition of gluconeogenesis. This was also linked to a reduction in glucose oxidation accompanied by reduced PHD activity and stimulation of ME1 and G6PD, favouring a shift from glucose- to fatty acid oxidation. The reduced plasma insulin/glucose ratio indicate that 1-triple TTA may improve glucose tolerance in rats.

## Introduction

The insulin resistance syndrome or the metabolic syndrome is a collection of risk factors characterized by elevated plasma triacylglycerol (TAG) and low-high-density lipoprotein-cholesterol (HDL-cholesterol), abdominal obesity, hypertension and elevated blood glucose. The metabolic syndrome is strongly linked to hyperinsulinemia, insulin resistance, glucose intolerance and/or type 2 diabetes mellitus, as well as dyslipidemia.

Given that mitochondrial function and mitochondrial fatty acid oxidation capacity seem to regulate TAG levels both in liver and plasma, we have synthesized 2-(tridec-12 -yn-1-ylthio)acetic acid (1-triple TTA) which target the mitochondria (1). Notably,1-triple TTA has the length as a palmitic acid, in which the beta-carbon is substituted with a sulphur atom. In addition, it has a triple bond at the omega -end making it resistant to both beta-oxidation and omega-degradation. We have previously shown that liver-specific mitochondrial proliferation induced by (1-triple TTA)-treatment strongly reduces liver and plasma TAG levels [1,2]. The 1-triple TTA-mediated clearance of plasma TAG involves increased mitochondrial fatty acid oxidation, and may also result from lowered apolipoprotein C-III levels with subsequent increased lipoprotein protein lipase activity, and a possible hepatic reuptake of very low-density lipoprotein, facilitating drainage of fatty acids to the liver for β-oxidation and production of ketone bodies as extrahepatic fuel [2]. Both lipid- and carbohydrate metabolism are regulated by processes taking place in the liver. Thus, the ability of 1-triple TTA to regulate hepatic energy status and mitochondrial function prompted us to investigate whether 1-triple TTA could modulate glucose homeostasis.

**Fig 1:**
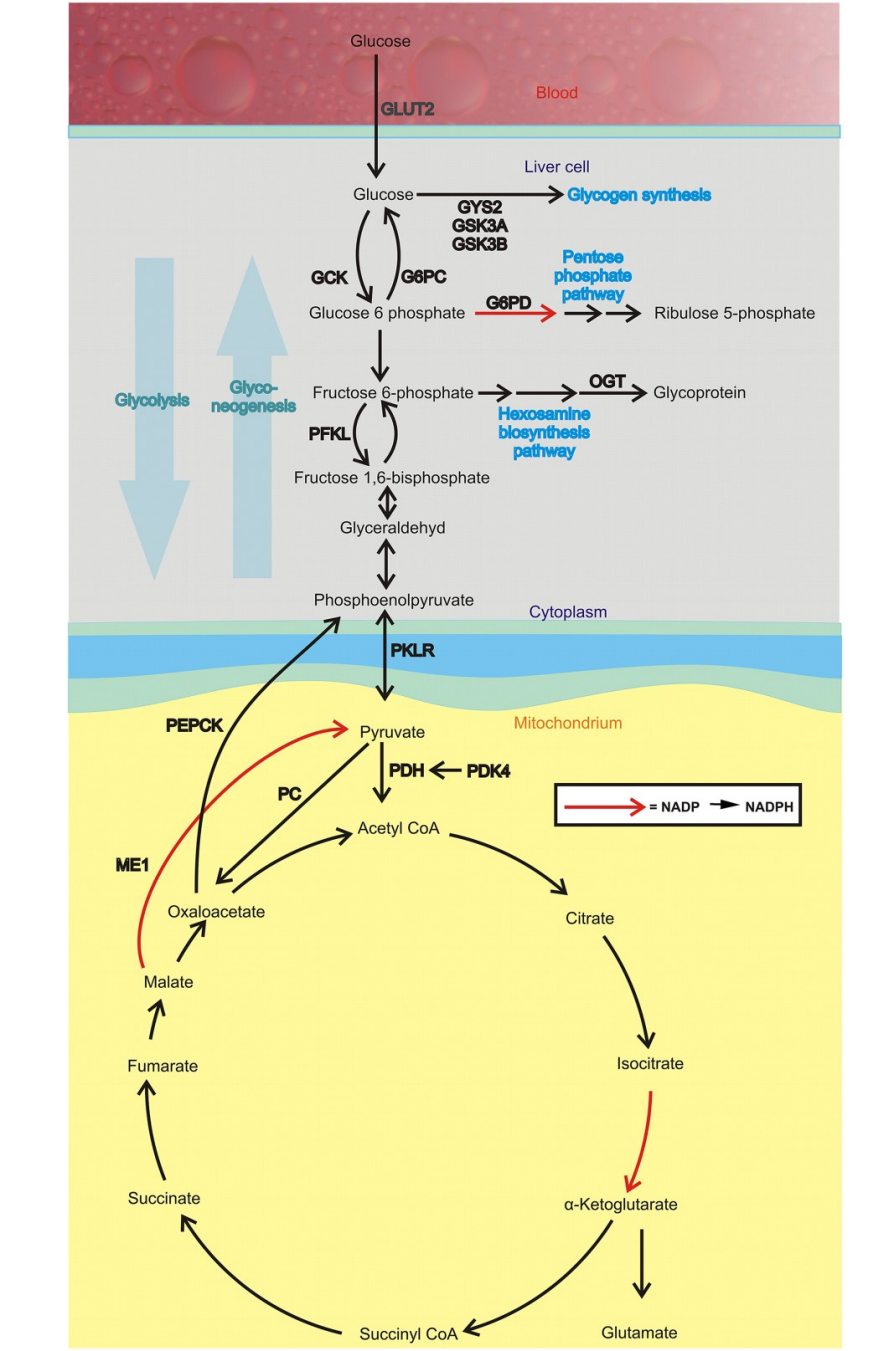
Glucose metabolism pathways in the liver. Abbreviations: glucose transporter 2 (GLUT2/*Slc2a2*); glucokinase phosphofructokinase (*Pfkl*); pyruvate kinase L/R (*Pklr*); pyruvate dehydrogenase kinase, isoenzyme 2 (*Pdk2*); pyruvate dehydrogenase kinase, isoenzyme 4 (*Pdk4*); pyruvate dehydrogenase kinase, isoenzyme 1 (*Pdk1*); pyruvate carboxylase (*Pc*); glucose-6 phosphatase (*G6pc*); glycogen synthetase 2 (*Gys2*); *Gys3a*; hepatic glycogen synthetase 3 beta (*Gsk3b*); glucose-6-phosphate dehydrogenase (*G6pd*); malic enzyme 1 (*Me1*); o-linked N-acetylglucosamine transferase (OGT).

Glucose homeostasis is physiologically maintained by the balance between glucose production by the liver and glucose utilization by peripheral tissues [3]. Moreover, liver is important to regulate plasma glucose levels during fasting and diabetic conditions both by degradation of its glycogen stores and through increased gluconeogenesis, and notably, increased fatty acid oxidation in the liver is generally thought to stimulate gluconeogenesis [4]. Phosphoenolpyruvate carboxykinase (PEPCK) and glucose-6-phosphatase catalyze the rate-limiting steps of gluconeogenesis (Fig 1) and the decarboxylation of oxaloacetate into phosphoenolpyruvate is obligate for mitochondrial-derived gluconeogenesis. Generally, this reaction has been attributed to the cytosolic isoform of PEPCK (PEPCK-C) although the mitochondrial isoform (PEPCK-M) could be involved in both normal and diabetic metabolism [5].

Gluconeogenesis can be considered the reversal of glycolysis [6] where glucose is converted to pyruvate (Fig 1). The last step in glycolysis is the conversion of phosphoenolpyruvate to pyruvate catalyzed by the rate-limiting enzyme pyruvate kinase. When glucose is abundant, i.e. during the fed state, glycolysis is active; glucose is converted to pyruvate in order to generate ATP and provide building blocks for cellular components. The pyruvate dehydrogenase (PDH) complex is a multi-enzyme complex regulating the conversion pyruvate to acetyl-CoA in the mitochondrial matrix. Reversely, the complex is inactivated during fasted states to conserve glucose and therefore shifts the cell´s energy supply from glucose to fatty acid oxidation [7].

The hexosamine biosynthesis pathway is a relatively minor branch of glycolysis. It utilizes fructose 6-phosphate as substrate, and encompasses approximately 3% of total glucose used. Elevated o-linked N-acetylglucosamine (OGT) levels implies increased hexosamine biosynthesis pathway-flux [8]. Several reports have proposed that flux through the hexosamine synthesis pathway may function as a cellular nutrient sensor and play a role in the development of insulin resistance and vascular complications of diabetes [8,9].

The pentose phosphate pathway is an anabolic pathway parallel to glycolysis, which generates pentoses and ribose-5 phosphate from glucose. The pathway is important for the synthesis of nucleotides and aromatic amino acids. Glucose 6-phosphate dehydrogenase is the rate limiting enzyme of this pathway, and the production of NADPH during this step in the oxidative phase of the pathway can be used in i.e. fatty acid synthesis [10].

While TAG stored in adipose tissue is considered the principle energy reserve in mammals, in addition, glucose is stored as glycogen in liver and skeletal muscle, for rapid mobilization during times of energy deficit. Moreover, muscle and liver glycogen deposition is decreased during insulin resistance and type 2 diabetes mellitus [11,12]. Elevated fasting plasma TAG is associated with dysregulation of glucose and as stimulated mitochondrial fatty acid oxidation is known to reduce plasma TAG (triacylglycerol), drugs that target mitochondrial function can potentially also improve glucose homeostasis and prevent type 2 diabetes [13–15].

In this study, we aimed to test how 1-triple TTA regulates plasma and hepatic glucose homeostasis, by studying key genes in hepatic glycolysis, gluconeogenesis, glycogen synthesis, the hexosamine biosynthesis pathway and the pentose phosphate pathway in rats and *in vitro* in human cell lines and primary cells.

## Materials and methods

### Animal study

The animal study was conducted according to the Guidelines for the Care and Use of Experimental Animals, in accordance with the Norwegian legislation and regulations governing experiments using live animals, and approved by the Norwegian State Board of Biological Experiments with Living Animals (Permit number 2015–7367). Male Wistar rats, *Rattus Norvegicus*, 5 weeks old, were purchased from Taconic (Ejby, Denmark). Upon arrival the rats were randomized using Research Randomizer [16], labeled and placed in open cages, four in each cage, where they were allowed to acclimatize to their surroundings for one week. During the acclimatization and experiment period, the rats had unrestricted access to chow and tap water and were kept in a 12 h light/dark cycle at a constant temperature (22 ± 2°C) with a relative humidity of 55% (± 5%). Upon start of the experiment, the rats were block randomized to their respective interventions. During the three weeks long experiment, there were two rats in each cage separated with a divider that let them have sniffing contact. All rats were given chow throughout the experiment. In addition, group 1, control (n = 8) received 0.5 ml 0.5% methylcellulose from the Hospital Pharmacy (Bergen, Norway) daily and group 2 (n = 6) received 100 mg/kg body weight 1-triple TTA (C_15_H_26_O_2_S, obtained from Synthetica AS, Oslo, Norway) dissolved in 0.5 ml 0.5% methylcellulose daily. Methylcellulose was given orally by gavage by a technician blinded to the experimental setup. All animals were weighed daily and feed intake was determined weekly.

At sacrifice, rats, equally divided between groups throughout the day, were anaesthetized by inhalation of 2–5% isoflurane (Schering-Plough, Kent, UK), the abdomen opened along the midline, and exsanguination was performed using cardiac puncture. EDTA-blood was collected and immediately chilled on ice. The samples were centrifuged and plasma was stored at—80°C prior to analysis. Liver was collected and weighed and a fresh sample from each liver was used for β-oxidation analysis. The remaining part of the liver was immediately snap-frozen in liquid nitrogen and stored at—80°C until further analysis.

### Plasma glucose and insulin

Glucose was measured enzymatically in EDTA-plasma on a Hitachi 917 system (Roche Diagnostics GmbH, Mannheim, Germany) using the GLUC2 kit from Roche Diagnostics. Insulin was measured using the Rat/Mouse insulin ELISA kit from Merck (Darmstadt, Germany).

### Pyruvate dehydrogenase activity in liver

Using TissueLyzer II (Qiagen, Hilden, Germany), approximately 100 mg frozen liver sample from each animal was homogenized in 500 μl PDH assay buffer from the pyruvate dehydrogenase activity assay kit (MAK 183, Sigma-Aldrich, St. Louis, MO, USA). Homogenates were centrifuged for 5 min at 10.000 x g at 4°C, the supernatant was removed and added 2 volumes of 4 M ammonium sulphate, and precipitation allowed to occur for 20 min on ice before re-centrifugation as above. The pellet was resuspended to the original volume using PDH assay buffer, and 2 μl per sample was used to analyse PDH activity according to the supplier’s manual. Protein was measured using the DC Protein Assay (Bio-Rad Laboratories, Hercules, CA, USA), and activity per mg protein was calculated.

### Hepatic glycogen, glucose, fructose 6-phosphate and NADPH

Using TissueLyzer II (Qiagen, Hilden, Germany), approximately 50 mg frozen liver sample from each animal was homogenized in 500 μl PBS (10%) at 2 min 25 Hz x 2, before sonication in a sonicator water bath for 30 sec with 15 sec pause x 3. After centrifugation at full speed for 5 min at 4°C, the supernatant was frozen in aliquots at -20°C. A 50 μl sample volume was used in the NADPH assay kit, and 10 ul of a 1:4 diluted homogenate was used in the fructose 6-phosphate assay kit according to the supplier’s manuals (both from Abcam, Cambridge, Great Britain). For measurement of glycogen and glucose, 10% homogenates in water were prepared using TissueLyzer as above, followed by 10 min at 100°C. Homogenates were centrifuged at 17.000 x g for 10 minutes at 4°Ca nd stored at -20°C. For the Abcam Glycogen Assay Kit, homogenates were diluted 1:100 and 30 μl sample volume was used. For the Abcam Glucose Assay Kit, homogenates were diluted 1:30 and 25 μl sample volume was used. For all homogenates, protein was measured using the DC Protein Assay (Bio-Rad Laboratories).

#### Hepatic palmitoyl-CoA oxidation

1 g fresh liver sample was chilled on ice and homogenized in 4 ml ice-cold sucrose medium (0.25 M sucrose, 10 mM HEPES, and 1 mM Na_4_EDTA, adjusted to a pH of 7.4 with KOH/HCl) as previously described [17]. The homogenates were centrifuged at 1030 RCF for 10 min at 4°C and the post-nuclear fraction was removed and used for further analysis. Palmitoyl-CoA oxidation was measured immediately in the post-nuclear fraction from fresh liver as acid-soluble products, as described [18]. The amount of protein was measured by the DC Protein Assay kit (Bio-Rad Laboratories).

### Hepatic gene expression analysis

Tissue samples (20 mg frozen liver) were homogenized in Rneasy Lysis Buffer from Qiagen (Cat.: 79216, Hilden, Germany) with 1% β-mercaptoethanol using Tissuelyser II (Qiagen) for 2x 2 min at 25 Hz, and total cellular RNA was further purified using the RNeasy mini kit (Qiagen, Hilden, Germany) including DNase digestion. 500 ng RNA was reverse transcribed using High Capacity cDNA Reverse Transcription Kits (Applied Biosystems, Waltham, Massachusetts, USA). qPCR was performed on Sarstedt 384-well Multiply-PCR plates (Sarstedt Inc., Newton, NC, USA) using ABI Prism 7900HT Sequence detection system from Applied Biosystems with the software SDS 2.3. Together with 2x Taqman buffer from Applied Biosystems, the following probes and primers from Applied Biosystems were used to detect mRNA levels of interests: Insulin receptor (*Insr*); glucose transporter 2 (GLUT2/*Slc2a2*); glucokinase phosphofructokinase (*Pfkl*); pyruvate kinase L/R (*Pklr*); pyruvate dehydrogenase kinase, isoenzyme 2 (*Pdk2*); pyruvate dehydrogenase kinase, isoenzyme 4 (*Pdk4*); pyruvate dehydrogenase kinase, isoenzyme 1 (*Pdk1*); phosphoenolpyruvate carboxykinase, cytosolic (PEPCK-C/*Pck1*); phosphoenolpyruvate carboxykinase-mitochondrial (PEPCK-M/*Pck2*); pyruvate carboxylase (*Pc*); hepatocyte nuclear factor 4a (*Hnf4a*); glucose-6 phosphatase (*G6pc*); glycogen synthetase 2 (*Gys2*); *Gys3a*; hepatic glycogen synthetase 3 beta (*Gsk3b*); glucose 6-phosphate dehydrogenase (*G6pd*); malic enzyme 1 (*Me1*); sterol regulatory element-binding protein 1 (*Srebp1*); stearoyl-CoA desaturase (*Scd-1*); MLC interacting protein like/ChREBP (*Mlxipl*); o-linked N-acetylglucosamine transferase (*Ogt*). Each probe was run with standard curve using either a representative cDNA sample or cDNA from universal rat reference RNA (URRR, Agilent). Expression levels were normalized to the average of the reference gene large ribosomal protein P0 (*36b4*, acc.no. M17885), and values relative to control are shown.

### Culturing of human myotubes

Multinucleated human myotubes were established by activation and proliferation of satellite cells isolated from a small biopsy (100–200 mg) of *musculus vastus lateralis* from four healthy men (age 21–29 years, weight 67–83 kg). The biopsies were obtained after informed written consent and approval by the Regional Committee for Medical and Health Research Ethics South East, Oslo, Norway (reference number: 2011/2207). Isolation of satellite cells was based on the method of Henry et al. [19], modified according to Gaster et al. [20,21], For proliferation of myoblasts DMEM-Glutamax^™^ (5.5 mM glucose) medium supplemented with 2% FBS and 2% Ultroser G was used. At approximately 80% confluence the culture medium was changed to DMEM-Glutamax^™^ (5.5 mM glucose) supplemented with 2% FBS and 25 pM insulin to initiate differentiation into multinucleated myotubes. The cells were differentiated for seven days. 1-triple TTA or vehicle (DMSO) were added 96 h before harvesting.

### Culturing of Huh7 cells

Human hepatoma Huh7 cells, purchased from ATCC (LGC Standards, Middlesex, UK), were grown in DMEM-Glutamax^™^ (5.5 mM glucose) medium supplemented with 10% FBS. 1-triple TTA or vehicle (DMSO) were added 48 h before harvesting.

### RNA isolation and analysis of gene expression by qPCR in Huh7 cells

Total RNA was isolated from cells using the RNeasy Mini Kit according to the supplier’s protocol. RNA was reversely transcribed with a High-Capacity cDNA Reverse Transcription Kit and TaqMan Reverse Transcription Reagents using a PerkinElmer 2720 Thermal Cycler (25°C for 10 min, 37°C for 80 min, 85°C for 5 min). Primers were designed using Primer Express^®^ (Applied Biosystems). qPCR was performed using a StepOnePlus Real-Time PCR system (Applied Biosystems). Target genes were quantified in duplicates carried out in a 25 μl reaction volume according to the supplier´s protocol. All assays were run for 44 cycles (95°C for 15 s followed by 60°C for 60 s). Expression levels were normalized to the average of the housekeeping gene large ribosomal protein P0 (*36B4*, acc.no. M17885). The following primers were used: 36B4; CD36 molecule (*CD36*, acc.no. L06850); cytochrome c-1 (*CycC*, acc.no. NM001916); pyruvate dehydrogenase kinase, isoenzyme 4 (*PDK4*, acc.no. BC040239); pyruvate kinase L/R (*PKLR*, acc.no NM000298); uncoupling protein 2 (*UCP2*, acc.no AF019409.1); uncoupling protein 3 (*UCP3*, acc.no. AF050113).

### Oxidation and uptake of glucose and fatty acids in Huh7 cells and cultured human myotubes

Cells were cultured on 96-well CellBIND^®^ microplates. [1-^14^C]oleic acid (18.5 kBq/ml), 100 μM, or D-[^14^C(U)]glucose (21.46 kBq/ml), 200 μM, were given during 4 h CO_2_ trapping as previously described [22]. In brief, a 96-well UniFilter^®^-96 GF/B microplate was mounted on top of the CellBIND^®^ culture plate and CO_2_ production was measured in DPBS medium with 10 mM HEPES and 1 mM L-carnitine adjusted to pH 7.2–7.3. CO_2_ production and cell-associated (CA) radioactivity were assessed using a 2450 MicroBeta^2^ scintillation counter (PerkinElmer). The sum of ^14^CO_2_ and CA radioactivity was taken as a measurement of total cellular uptake of substrate. Protein content in each well was measured according to Bradford [23].

### Glycogen synthesis in Huh7 cells

Huh7 cells grown in 12-well plates were incubated in DMEM containing 5.5 mM glucose supplemented with D-[^14^C(U)]-glucose (37 kBq/ml) as described [24] for 4 h. Briefly, after washing cells twice with ice-cold phosphate buffered saline (PBS), cells were exposed to 1 M potassium hydroxide (KOH) for solubilization. Afterwards, samples were incubated with a final concentration of 3 M KOH and 20 mg/ml glycogen and heated at 80°C for 20 min. Glycogen precipitates were received by adding ice-cold absolute ethanol, washed once with 70% ethanol and dissolved in 500 μl distilled water. Incorporated D-[^14^C(U)]-glucose was assessed by liquid scintillation counting. Protein content was measured according to Bradford [23].

### Glucose output in Huh7 cells

Huh7 cells cultured in 12-well plates were incubated in DMEM containing 5.5 mM glucose supplemented with D-[^14^(U)]-glucose (37 kBq/ml) for 4 h. After washing twice with PBS, the cells were incubated in DPBS medium with 10 mM HEPES without glucose. An aliquot of the medium was withdrawn after 0.5, 1, 1.5, 2, 2.5, 3, 4, 4.5 and 5 h and the radioactivity assessed by liquid scintillation counting. Protein content in each well was measured according to Bradford [23].

### Statistical analysis

Statistical difference was analyzed using Student’s t-test on the primary outcomes; glucose and insulin levels, and the secondary outcome;s hepatic gene expression and enzyme activities. P-values < 0.05 were considered significant. The results are shown as means ± standard deviation (SD) of 5–8 rats per group, or means ± SEM for cell culture analyses. Pearson’s correlation coefficients were used when comparing two independent variables. The statistics was performed using IBM SPSS Statistics for Windows, Version 22.0 (IBM Corp. Armonk, USA) and graphs were designed with GraphPad Prism for Windows, Version 6.00 (GraphPad Software, La Jolla, CA, USA).

## Results

### 1-triple TTA affected hepatic mitochondrial function and decreased insulin in plasma

**Fig 2:**
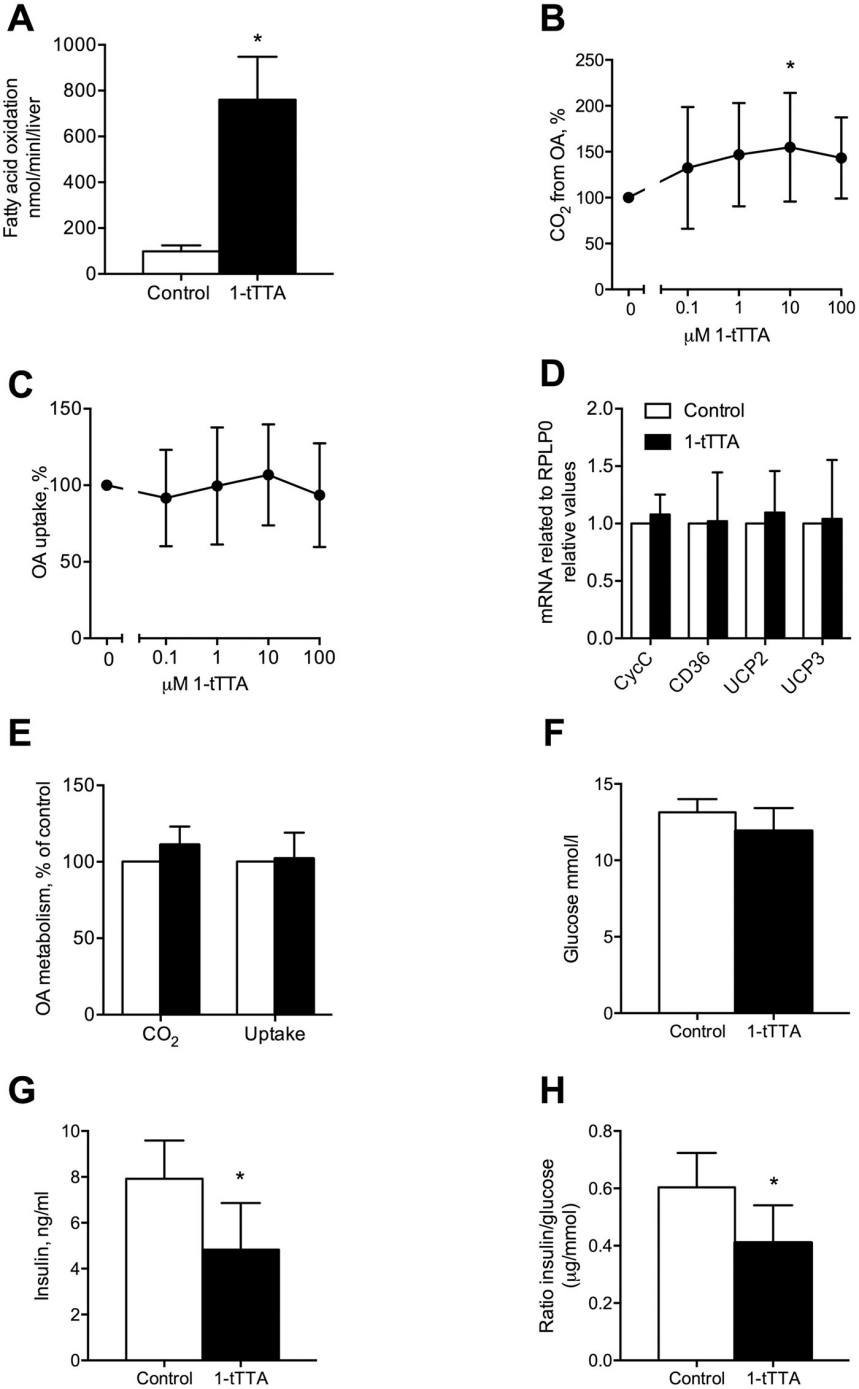
Effects 1-triple TTA (1-tTTA) on fatty acid metabolism and plasma insulin and glucose levels both in vivo and in vitro. (**a**) Rats were treated with vehicle (control) or 100 mg/kg body weight 1-tTTA for 3 weeks and palmitoyl-CoA oxidation in liver was measured (n = 5–8). (**b**) Oleic acid oxidation and (**c**) oleic acid uptake, measured as described in Materials and Methods, in human Huh7 liver cells treated with vehicle (control) or 1-triple-TTA for 48 h (n = 6–8). (**d**) Gene expression of cytochrome c-1 (*CycC*), CD36 molecule (*CD36*), uncoupling protein (*UCP*)*2* and *UCP3* in Huh7 cells treated with 10 μM 1-tTTA for 48 h (n = 6–8). (**e**) Oleic acid oxidation and uptake in human myotubes treated with 10 μM 1-tTTA for 96 h (n = 4). (**f**) Plasma glucose, (**g**) insulin, and (**h**) insulin/glucose ratio in rats treated with vehicle (control) or 1-tTTA for 3 weeks (n = 5–8). Values shown are means with standard deviation. Statistically significant differences between means were determined using Students t-test, *p < 0.05 vs. control.

Three weeks of 1-triple TTA treatment in rats increased hepatic fatty acid oxidation (Fig 2A), but did not affect weight gain or food intake. In Huh7 liver cells, the 1-triple TTA-induced increase in oxidation of oleic acid (Fig 2B) was associated with unchanged fatty acid uptake (Fig 2C), and gene expression of *CycC*, *CD36*, *UCP2* and *UCP3* remained unaltered (Fig 2D). No increased fatty acid oxidation was found in cultured human skeletal muscle cells (Fig 2E), suggesting that the effect of 1-triple TTA is cell type specific. This is in line with findings demonstrating no change in energy state in skeletal muscle from 1-triple TTA-treated rats, whereas in liver a lowered energy state was found reflected by an increased AMP/ATP-ratio [2].

The liver is important for the regulation of the plasma glucose level, and thus 1-triple TTA as a liver targeted compound could potentially influence plasma glucose and insulin. Treatment of rats with 1-triple TTA for 21 days tended to lower the plasma glucose level compared to control (Fig 2F, p = 0.076). The plasma insulin level was significantly reduced in the 1-triple TTA treated group (Fig 2G). The lower insulin/glucose ratio in the 1-triple TTA treated rats is indicative of insulin sensitization after 1-triple TTA-treatment (Fig 2H).

### 1-triple-TTA changed hepatic glycolysis, gluconeogenesis and glycogen synthesis

**Fig 3:**
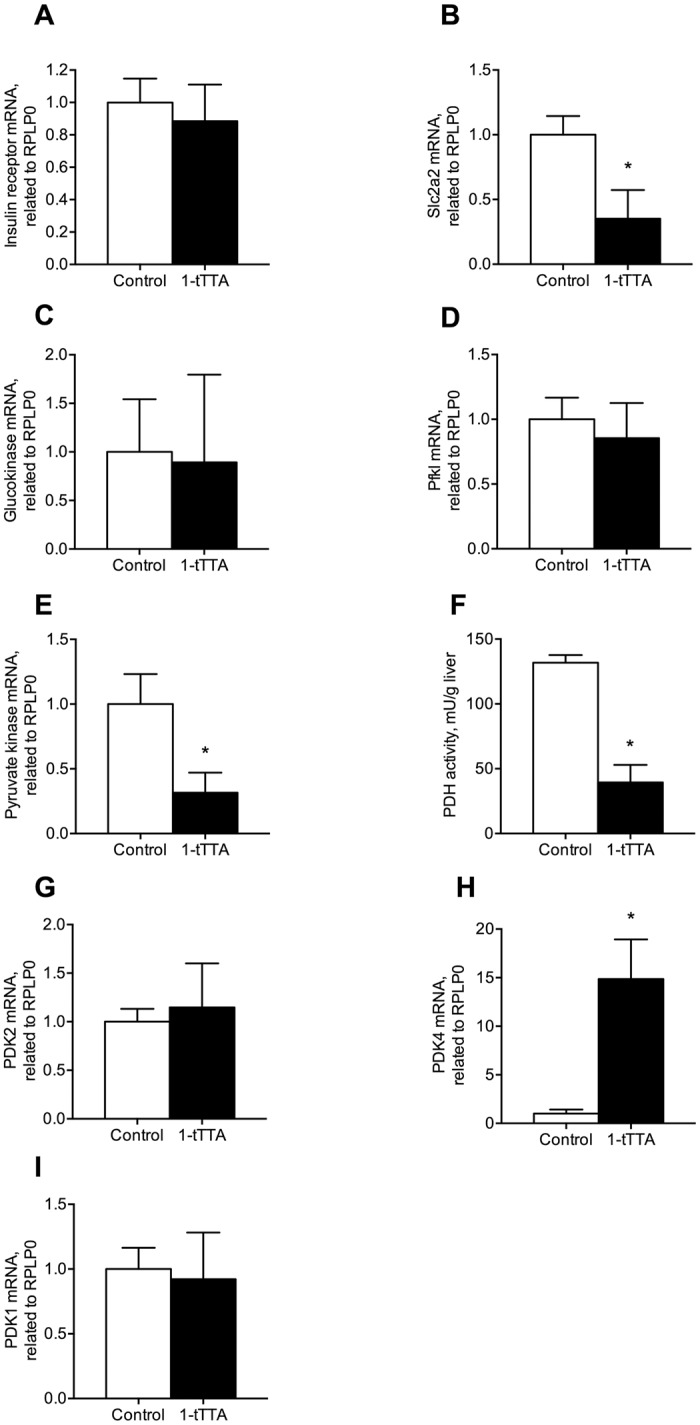
Hepatic mRNA levels and activity of enzymes involved in glycolysis after 3 weeks treatment with vehicle (control) or 1-triple TTA (1-tTTA) in rats. (**a**) Insulin receptor mRNA level, (**b**) *Slc2a2* mRNA level, (**c**) glucokinase mRNA level, (**d**) phosphofructokinase (*Pfkl*) mRNA level, (**e**) pyruvate kinase mRNA level, (**f**) pyruvate dehydrogenase (PDH) complex activity, (**g**) pyruvate dehydrogenase kinase, isoenzyme 2 (*Pdk2*) mRNA level, (**h**) pyruvate dehydrogenase kinase, isoenzyme 4 (*Pdk4*) mRNA level, (**i**) pyruvate dehydrogenase kinase, isoenzyme 1 (*Pdk1*) mRNA level. Gene expression levels were normalized to the house keeping gene *Rplp0* and relative values are given. All values shown are means with standard deviation (n = 6–8). Statistically significant differences between means were determined using Students t-test, *p < 0.05 vs. control.

1-triple TTA could potentially influence the homeostatic control of blood glucose and insulin action through its hepatic effects. This prompted us to investigate key genes in glycolysis, gluconeogenesis and glycogen synthesis in liver. Three weeks of 1-triple TTA-treatment in rats did not alter hepatic gene expression of insulin receptor (*Insr*), however, the mRNA levels of glucose transporter 2 (*Slc2a2*), was significantly decreased in liver by 1-triple TTA (Fig 3A and 3B). While hepatic gene expression of glucokinase and phosphofructokinase (*Pfkl*) were unchanged (Fig 3C and 3D), pyruvate kinase (*Pklr*) and the activity of PDH were significantly decreased by 1-triple TTA (Fig 3E and 3F). PDH is a mitochondrial multiplex enzyme and one of the major regulators of carbohydrate fuel homeostasis in mammals. It is regulated by a phosphorylation/dephosphorylation cycle, and in line with the decreased PDH activity (Fig 3F), gene expression of one of its major regulators, *Pdk4*, was markedly increased (10-15-fold) by 1-triple TTA (Fig 3H). In contrast, the hepatic gene expression of other kinases involved in PDH inactivation, *Pdk1* and *Pdk2*, remained unaltered after 1-triple TTA-administration (Fig 3G and 3I).

**Fig 4:**
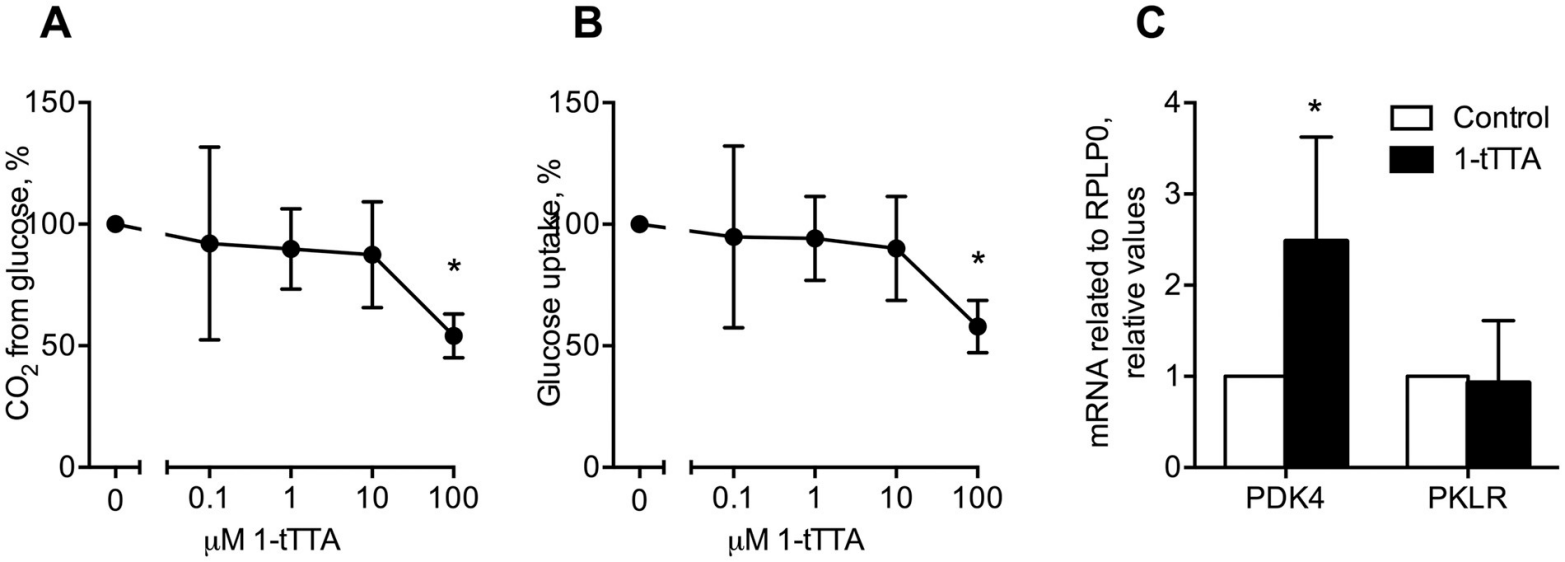
Effects 1-triple TTA (1-tTTA) on glucose metabolism and gene expression in Huh7 cells. Huh7 cells were treated with vehicle (control) or 1-tTTA for 48 h. (**a**) Glucose oxidation. (**b**) glucose uptake. (**c**) mRNA expression of pyruvate dehydrogenase kinase, isoenzyme 4 (*PDK4*) and pyruvate kinase L/R (*PKLR*) after treatment with 10 μM 1-tTTA. Gene expression levels were normalized to the house keeping gene RPLP0 and relative values are given. A, B: n = 5, C: n = 4–8. Statistically significant differences between means were determined using Students t-test, *p < 0.05 vs. control.

Noteworthy, in human Huh7 liver cells, the highest concentration of 1-triple TTA (100 μM) reduced glucose oxidation and glucose uptake (Fig 4A and 4B). These findings were associated with a significantly increased gene expression of *Pdk4* whereas the pyruvate kinase (*Pklr*) expression remained unchanged (Fig 4C).

**Fig 5:**
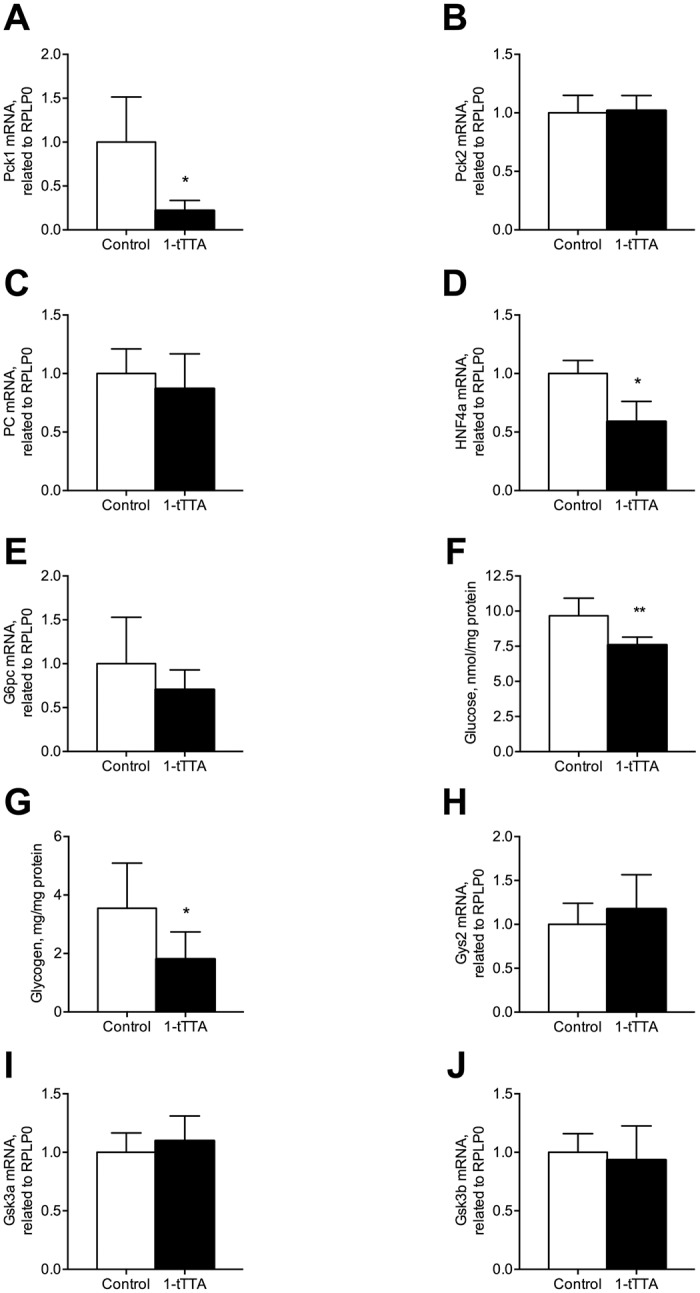
Hepatic glucose and glycogen levels, and mRNA levels of enzymes involved in gluconeogenesis and glycogen synthesis after 3 weeks treatment with vehicle (control) or 1-triple TTA (1-tTTA) in rats. (**a**) Phosphoenolpyruvate carboxykinase, cytosolic (PEPCK-C/*Pck1*) mRNA level, (**b**) phosphoenolpyruvate carboxykinase-mitochondrial (PEPCK-M/*Pck2*) mRNA level, (**c**) pyruvate carboxylase (*Pc*) mRNA level, (**d**) hepatocyte nuclear factor 4a (*Hnf4a*) mRNA level, (**e**) glucose-6 phosphatase (*G6pc*) mRNA level, (**f**) the hepatic glucose level, (**g**) the hepatic glycogen level, (**h**) glycogen synthetase 2 (*Gys2*) mRNA level, (**i**) *Gys3a* mRNA level, (**j**) glycogen synthetase 3 beta (*Gsk3b*) mRNA level. Gene expression levels were normalized to the house keeping gene RPLP0 and relative values are given. All values shown are means with standard deviation (n = 6–8). Statistically significant differences between means were determined using Students t-test, *p < 0.05 vs. control.

**Fig 6:**
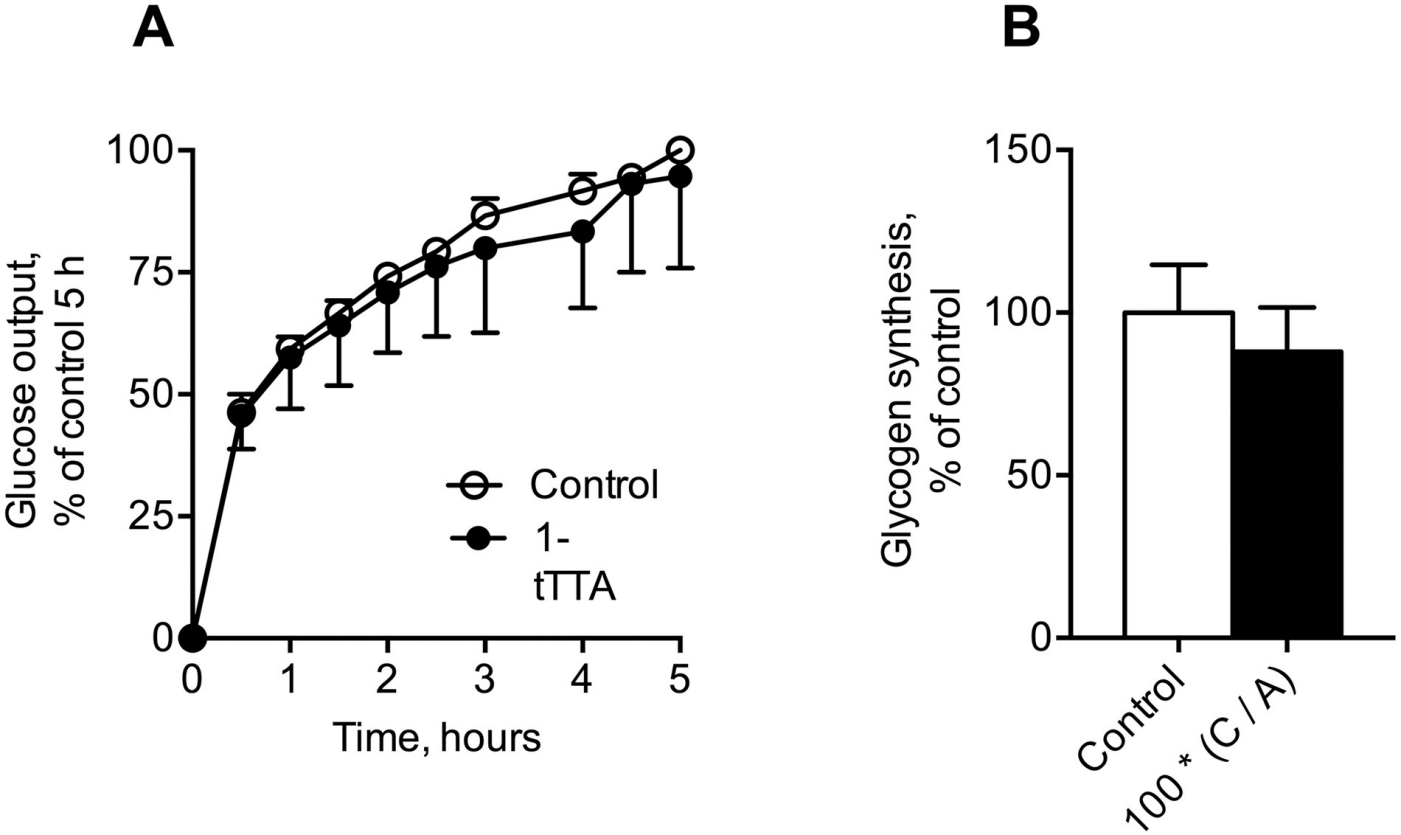
Effects 1-triple TTA (1-tTTA) on glucose output (a) and glycogen synthesis (b) in Huh7 cells. Huh7 cells were treated with vehicle (control) or 10 μM 1-tTTA for 48 h. Glucose output and glycogen synthesis were measured as described in Materials and Methods. All values shown are means with standard deviation (n = 3).

We next investigated whether 1-triple TTA caused changes in mRNA expression of key genes in gluconeogenesis and glycogen synthesis and -formation in rats. 1-triple TTA significantly reduced gene expression of *Pck1* (encoding PEPCK-C), whereas the mRNA level of *Pck2* (PEPCK-M) remained unaltered (Fig 5A and 5B). The mRNA level of pyruvate carboxylase (*PC*), the first regulatory enzyme of gluconeogenesis catalyzing the reaction of pyruvate to oxaloacetate, remained unchanged after 1-triple TTA treatment (Fig 5C). However, the gene expression of hepatocyte nuclear factor 4a (*Hnf4a*), shown to regulate expression of PC [25], was significantly reduced by 1-triple TTA (Fig 5D). Moreover, the gene expression of glucose-6 phosphatase (*G6pc*) remained unaltered in the 1-triple TTA treated animal group (Fig 5E). Decreased expression of genes involved in gluconeogenesis seemed to be associated with decreased levels of liver glycogen and glucose (Fig 5F and 5G). However, 1-triple TTA did not change the gene expression of glycogen synthetase 2 (*Gys2*), glycogen synthetase kinase 3 alpha (*Gsk3a*) or the mRNA level of hepatic glycogen synthetase 3 beta (*Gsk3b*) (Fig 5H–5J). Moreover, glucose release and glycogen synthesis in Huh7 cells were insignificantly decreased by 4 days of 1-triple TTA treatment (Fig 6).

### 1-triple TTA changes the pentose phosphate pathway but not the hexosamine biosynthesis pathway

**Fig 7:**
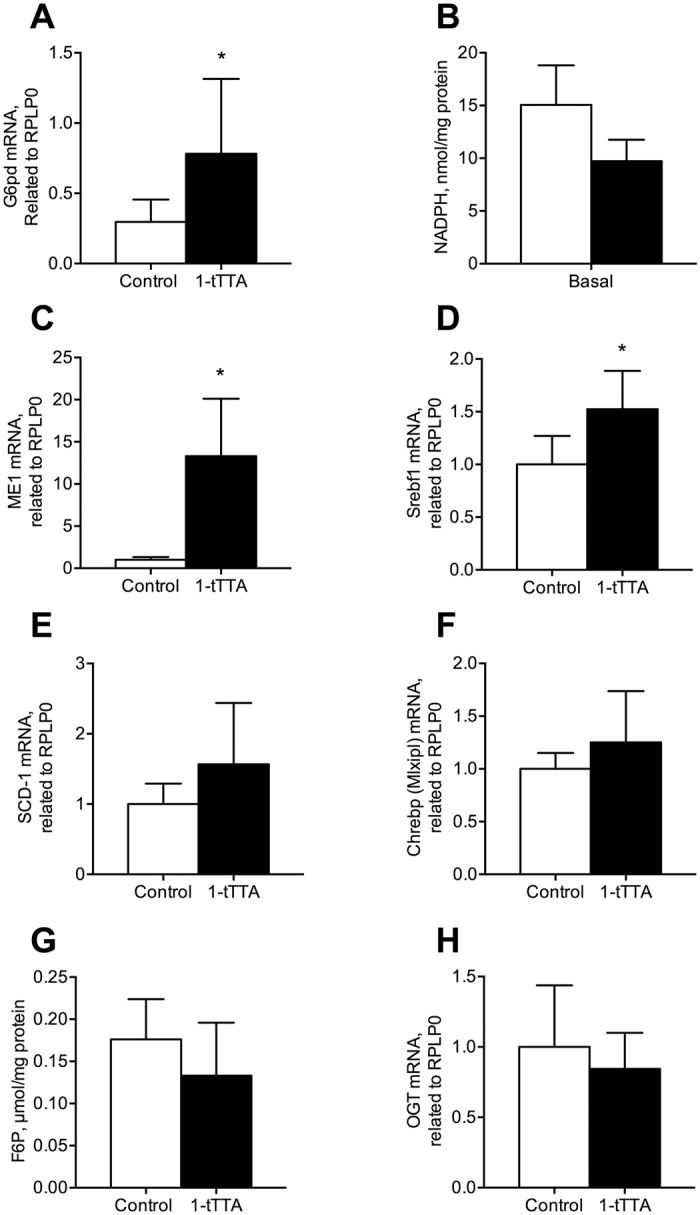
Hepatic NADPH level and expression of genes involved in the pentose phosphate pathway and the hexosamine pathway after 3 weeks treatment with vehicle (control) or 1-triple TTA (1-tTTA) in rats. (**a**) glucose-6-phosphate dehydrogenase (*G6pd*) mRNA level, (**b**) NADPH level, (**c**) malic enzyme (*Me*) mRNA level, (**d**) sterol regulatory element-binding protein 1 (*Srebp1*) mRNA level, (**e**) stearoyl-CoA desaturase (*Scd-1*) mRNA level, (**f**) ChREBP (*Mlxipl*) mRNA level, (**g**) fructose-6-phosphate (F6P) level, (**h**) o-linked N-acetylglucosamine transferase (*Ogt*) mRNA level. Gene expression levels were normalized to the house keeping gene RPLP0 and relative values are given. All values shown are means with standard deviation (n = 6–8). Statistically significant differences between means were determined using Student’s t-test, *p < 0.05 vs. control.

The pentose phosphate pathway is a metabolic pathway parallel to glycolysis, with glucose 6-phosphate dehydrogenase as the rate-limiting enzyme. Noteworthy, in rats treated with 1-triple TTA, increased gene expression of glucose 6-phosphate dehydrogenase (Fig 7A) was associated with an insignificant decrease in the hepatic level of NADPH (Fig 7B). Moreover, the mRNA level of malic enzyme (*Me1*), which generates NADPH for i.e. fatty acid biosynthesis, was significantly increased (10-15-fold) by 1-triple TTA in rats (Fig 7C). The sterol regulatory element-binding protein 1 (SREBP-1) represents a master regulator of biosynthesis of lipids from i.e. glucose [26]. 1-triple TTA increased gene expression of *Srebf1*, whereas gene expression of stearoyl-CoA desaturase (*Scd-1*) remained unchanged (Fig 7D and 7E). In the liver, the carbohydrate-responsive element-binding protein (ChREBP) mediates activation of several regulatory enzymes of glycolysis and lipogenesis [27]. The hepatic gene expression of ChREBP (*Mlxipl*) tended to increase by 1-triple TTA, but this was not statistically significant (Fig 7F).

Fructose 6-phosphate (F6P), which is the entry substrate for the hexosamine biosynthesis pathway, remained unchanged after 1-triple TTA-administration in rats (Fig 7G). Similarly, the gene expression of o-linked N-acetylglucosamine transferase (*Ogt*), important in regulation the flux in the hexosamine biosynthesis pathway, was unaffected by 1-triple TTA-administration (Fig 7H).

## Discussion

This work demonstrates that the hypolipidemic mitochondria-targeted compound 1-triple TTA significantly reduced liver glycogen and glucose content and also tended to reduce plasma glucose (p < 0.076) levels in rats. Indeed, the plasma insulin level was significantly reduced accompanied by a reduced plasma insulin/glucose ratio. This was linked to suppression of gluconeogenesis, most likely combined with inhibition of glucose utilization through reduced activity of PDH and increased expression of PDK4. These findings were supported by observations of lowered glucose oxidation in human Huh7 liver cells treated with 1-triple TTA, and of increased liver-specific fatty acid oxidation in rats. Altogether, this indicates that 1-triple TTA has potential as an antidiabetic drug.

The liver is one of the main organs for glucose storage and it plays a crucial role in blood glucose regulation and indeed hepatic glucose production has been shown to be important in the development of hyperglycemia in diabetes mellitus patients [28]. Moreover, it has been reported that increased gluconeogenesis in the liver results in glucose intolerance in animals models [29], and in most animal models of diabetes expression levels of PEPCK are elevated [30]. PEPCK, which is one of the rate-limiting enzymes of gluconeogenesis, is regulated by different hormones at the transcription level. Accordingly, suppression of PEPCK gene expression (*Pck1* and *Pck2*) is a potential target for mitochondrial proliferating antidiabetic drugs. Administration of 1-triple TTA strongly decreased gene expression of *Pck1*, whereas the mRNA level of *Pck2* remained unaltered compared to control animals. A role for mitochondrial PEPCK in the regulation of hepatic gluconeogenesis has been suggested [5,31]. However, our results indicate that the lowering of the insulin/glucose ratio observed with 1-triple TTA is mediated through the cytosolic isoform of PEPCK (PEPCK-C) and not the mitochondrial isoform (PEPCK-M).

The next question raised is how 1-triple TTA suppressed *Pck1* gene expression. In the present study 1-triple TTA had no effect on the mRNA level of glucokinase (Fig 3C) and we have previously reported that 1-triple TTA did not change AMPK phosphorylation [2]. Thus, the effect of 1-triple TTA on PEPCK–C gene expression seemed not to be mediated through the AMPK pathway. Interestingly, HNF4 is reported to be a regulator of both PEPCK and pyruvate kinase. In the present study in rats, gene expression of *Hnf4a* was decreased in parallel with a decreased mRNA level of *Pck1*. Thus, decreased expression of *Hnf4a* mRNA would be responsible for a lower amount of *Pepck* and *Pck1* mRNA. HNF4 promotes *Chrebp* gene transcription in response to glucose and the target genes of CHREBP are involved in glycolysis, gluconeogenesis and lipogenesis. Moreover, glucose and insulin coordinately regulate *de novo* lipogenesis from glucose in the liver and insulin activates several transcription factors including SREBP-1C. Interestingly, 1-triple TTA increased the gene expression of *Srebf1* and tended to increase the mRNA levels of *Chrebp* as well as *Scd-1*, encoding a desaturase catalyzing an important step in lipogenesis.

PDH, located in the mitochondrial matrix, is the first component enzyme of the pyruvate dehydrogenase complex, which contributes to transforming pyruvate into acetyl-CoA, which can be used in the citric acid cycle and subsequent cellular respiration. Thus, PDH contributes to linking the glycolysis metabolic pathway to the citric acid cycle and the release of energy via NADH. PDK4 is also located in the mitochondrial matrix and inhibits the pyruvate dehydrogenase complex by phosphorylating one of its subunits, thereby contributing to the regulation of glucose homeostasis. This is particularly important during starvation, where inhibition of PDH blocks the oxidation of glucose as well as gluconeogenetic substrates originating from muscle breakdown [32]. Thus, glucose is spared for use in the brain, and muscle breakdown is prevented during prolonged starvation. Interestingly, 1-triple TTA dramatically decreased PDH activity accompanied by a strong increase in gene expression of *Pdk4*. This is in line with reduced PDH activity and reduced oxidation of glucose. Noteworthy, the mRNA levels of *Pdk1* and *Pdk2* were not affected by 1-triple TTA, indicating that they are less important during inhibition of PDH activity by 1-triple TTA. Induced expression of *Pdk4* in Huh7 cells support a specific effect of 1-triple TTA on PDH activity.

The hexosamine biosynthesis pathway is a relative minor branch of glycolysis, and 1-triple TTA had no effect on the hepatic fructose 6-phosphate level or the gene expression of *Ogt*. The pentose phosphate pathway does not involve oxidation of glucose, and its primary role is anabolic rather than catabolic. Interestingly, 1-triple TTA increased the gene expression of glucose 6-phosphate dehydrogenase accompanied by unchanged liver levels of NADPH. One of the uses of NADPH in the cells is to prevent oxidative stress, and in addition the production of ribose-5 phosphate by the pentose phosphate pathway is used in the synthesis of nucleotides and nucleic acids. Noteworthy, fatty acid analogues containing a S-atom in the three-position from the carboxyl end, i.e. tetradecylthioacetic acid (TTA), are reported to increase the redox state and decrease inflammation [33–36]. Moreover, TTA and 1-triple TTA stimulate mitochondrial biosynthesis accompanied by an increased amount of mitochondrial DNA [2].

In conclusion, decreased gluconeogenesis, mediated by down-regulation of PEPCK–C and pyruvate kinase accompanied by reduced pyruvate dehydrogenase activity, indicate plasma and liver glucose-lowering potential with the mitochondrially targeted fatty acid analogue 1-triple TTA in rats. The regulation of the PDH activity level by increased gene expression of pyruvate dehydrogenase kinase 4 was further supported by similar observations in human liver cells treated with 1-triple-TTA. Upregulation of the pentose phosphate pathway seemed to run in parallel with reduced glycolysis. Further studies are required to evaluate the potential use of 1-triple TTA to target pathogenic mechanism in disorders such as insulin resistance and type 2 diabetes in humans.

## References

[1] Berge RK, Tronstad KJ, Berge K, Rost TH, Wergedahl H, Gudbrandsen OA, et al (2005) The metabolic syndrome and the hepatic fatty acid drainage hypothesis. Biochimie. 87(1):15–20. 10.1016/j.biochi.2004.11.011 15733731

[2] Lindquist C, Bjorndal B, Rossmann CR, Tusubira D, Svardal A, Rosland GV, et al (2017) Increased hepatic mitochondrial FA oxidation reduces plasma and liver TG levels and is associated with regulation of UCPs and APOC-III in rats. Journal of Lipid Research. 58(7):1362–1373. 10.1194/jlr.M074849 28473603 PMC5496034

[3] Saltiel AR, Kahn CR (2001) Insulin signalling and the regulation of glucose and lipid metabolism. Nature. 414(6865):799–806. 10.1038/414799a 11742412

[4] Consoli A (1992) Role of liver in pathophysiology of NIDDM. Diabetes Care. 15(3):430–441. 10.2337/diacare.15.3.430 1559410

[5] Stark R, Guebre-Egziabher F, Zhao X, Feriod C, Dong J, Alves TC, et al (2014) A role for mitochondrial phosphoenolpyruvate carboxykinase (PEPCK-M) in the regulation of hepatic gluconeogenesis. J Biol Chem. 289(11):7257–7263. 10.1074/jbc.C113.544759 24497630 PMC3953244

[6] Pilkis SJ, Granner DK (1992) Molecular physiology of the regulation of hepatic gluconeogenesis and glycolysis. Annu Rev Physiol. 54 885–909. 10.1146/annurev.ph.54.030192.004321 1562196

[7] Zhang S, Hulver MW, McMillan RP, Cline MA, Gilbert ER (2014) The pivotal role of pyruvate dehydrogenase kinases in metabolic flexibility. Nutr Metab (Lond). 11(1):10 10.1186/1743-7075-11-10 24520982 PMC3925357

[8] Buse MG (2006) Hexosamines, insulin resistance, and the complications of diabetes: current status. Am J Physiol Endocrinol Metab. 290(1):E1–E8. 10.1152/ajpendo.00329.2005 16339923 PMC1343508

[9] Schleicher ED, Weigert C (2000) Role of the hexosamine biosynthetic pathway in diabetic nephropathy. Kidney Int Suppl. 77 S13–18. 10997685 10.1046/j.1523-1755.2000.07703.x

[10] Barcia-Vieitez R, Ramos-Martinez JI (2014) The regulation of the oxidative phase of the pentose phosphate pathway: new answers to old problems. IUBMB Life. 66(11):775–779. 10.1002/iub.1329 25408203

[11] Shulman GI, Rothman DL, Jue T, Stein P, DeFronzo RA, Shulman RG (1990) Quantitation of muscle glycogen synthesis in normal subjects and subjects with non-insulin-dependent diabetes by 13C nuclear magnetic resonance spectroscopy. N Engl J Med. 322(4):223–228. 10.1056/NEJM199001253220403 2403659

[12] Tomiyasu M, Obata T, Nishi Y, Nakamoto H, Nonaka H, Takayama Y, et al (2010) Monitoring of liver glycogen synthesis in diabetic patients using carbon-13 MR spectroscopy. Eur J Radiol. 73(2):300–304. 10.1016/j.ejrad.2008.10.019 19058940

[13] Harper P, Wadstrom C, Cederblad G (1993) Carnitine measurements in liver, muscle tissue, and blood in normal subjects. Clin Chem. 39(4):592–599. 8472351

[14] Reuter SE, Evans AM, Chace DH, Fornasini G (2008) Determination of the reference range of endogenous plasma carnitines in healthy adults. Ann Clin Biochem. 45(Pt 6):585–592. 10.1258/acb.2008.008045 18782814

[15] Reuter SE, Evans AM, Faull RJ, Chace DH, Fornasini G (2005) Impact of haemodialysis on individual endogenous plasma acylcarnitine concentrations in end-stage renal disease. Ann Clin Biochem. 42(Pt 5):387–393. 10.1258/0004563054889954 16168195

[16] Urbaniak G, Plous S: Research Randomizer. In, 2013.

[17] Berge RK, Flatmark T, Osmundsen H (1984) Enhancement of long-chain acyl-CoA hydrolase activity in peroxisomes and mitochondria of rat liver by peroxisomal proliferators. Eur J Biochem. 141(3):637–644. 10.1111/j.1432-1033.1984.tb08239.x 6146524

[18] Willumsen N, Hexeberg S, Skorve J, Lundquist M, Berge RK (1993) Docosahexaenoic acid shows no triglyceride-lowering effects but increases the peroxisomal fatty acid oxidation in liver of rats. J Lipid Res. 34(1):13–22. 8445337

[19] Henry RR, Abrams L, Nikoulina S, Ciaraldi TP (1995) Insulin action and glucose metabolism in nondiabetic control and NIDDM subjects. Comparison using human skeletal muscle cell cultures. Diabetes. 44(8):936–946. 10.2337/diab.44.8.936 7622000

[20] Gaster M, Beck-Nielsen H, Schroder HD (2001) Proliferation conditions for human satellite cells. The fractional content of satellite cells. APMIS. 109(11):726–734. 10.1034/j.1600-0463.2001.d01-139.x 11900051

[21] Gaster M, Kristensen SR, Beck-Nielsen H, Schroder HD (2001) A cellular model system of differentiated human myotubes. APMIS. 109(11):735–744. 10.1034/j.1600-0463.2001.d01-140.x 11900052

[22] Wensaas AJ, Rustan AC, Lovstedt K, Kull B, Wikstrom S, Drevon CA, et al (2007) Cell-based multiwell assays for the detection of substrate accumulation and oxidation. J Lipid Res. 48(4):961–967. 10.1194/jlr.D600047-JLR200 17213484

[23] Bradford MM (1976) A rapid and sensitive method for the quantitation of microgram quantities of protein utilizing the principle of protein-dye binding. Anal Biochem. 72 248–254. 10.1006/abio.1976.9999 942051

[24] Hessvik NP, Bakke SS, Fredriksson K, Boekschoten MV, Fjorkenstad A, Koster G, et al (2010) Metabolic switching of human myotubes is improved by n-3 fatty acids. J Lipid Res. 51(8):2090–2104. 10.1194/jlr.M003319 20363834 PMC2903803

[25] Chavalit T, Rojvirat P, Muangsawat S, Jitrapakdee S (2013) Hepatocyte nuclear factor 4alpha regulates the expression of the murine pyruvate carboxylase gene through the HNF4-specific binding motif in its proximal promoter. Biochim Biophys Acta. 1829(10):987–999. 10.1016/j.bbagrm.2013.05.001 23665043

[26] Xu HF, Luo J, Zhao WS, Yang YC, Tian HB, Shi HB, et al (2016) Overexpression of SREBP1 (sterol regulatory element binding protein 1) promotes de novo fatty acid synthesis and triacylglycerol accumulation in goat mammary epithelial cells. J Dairy Sci. 99(1):783–795. 10.3168/jds.2015-9736 26601584

[27] Iizuka K (2017) The Role of Carbohydrate Response Element Binding Protein in Intestinal and Hepatic Fructose Metabolism. Nutrients. 9(2). 10.3390/nu9020181 28241431 PMC5331612

[28] Morral N (2003) Novel targets and therapeutic strategies for type 2 diabetes. Trends Endocrinol Metab. 14(4):169–175. 12714277 10.1016/s1043-2760(03)00031-6

[29] Chakravarty K, Cassuto H, Reshef L, Hanson RW (2005) Factors that control the tissue-specific transcription of the gene for phosphoenolpyruvate carboxykinase-C. Crit Rev Biochem Mol Biol. 40(3):129–154. 10.1080/10409230590935479 15917397

[30] Mosseri R, Waner T, Shefi M, Shafrir E, Meyerovitch J (2000) Gluconeogenesis in non-obese diabetic (NOD) mice: in vivo effects of vandadate treatment on hepatic glucose-6-phoshatase and phosphoenolpyruvate carboxykinase. Metabolism. 49(3):321–325. 10.1016/s0026-0495(00)90132-x 10726908

[31] Stark R, Kibbey RG (2014) The mitochondrial isoform of phosphoenolpyruvate carboxykinase (PEPCK-M) and glucose homeostasis: has it been overlooked? Biochim Biophys Acta. 1840(4):1313–1330. 10.1016/j.bbagen.2013.10.033 24177027 PMC3943549

[32] Jeoung NH, Wu P, Joshi MA, Jaskiewicz J, Bock CB, Depaoli-Roach AA, et al (2006) Role of pyruvate dehydrogenase kinase isoenzyme 4 (PDHK4) in glucose homoeostasis during starvation. Biochem J. 397(3):417–425. 10.1042/BJ20060125 16606348 PMC1533314

[33] Muna ZA, Bolann BJ, Chen X, Songstad J, Berge RK (2000) Tetradecylthioacetic acid and tetradecylselenoacetic acid inhibit lipid peroxidation and interact with superoxide radical. Free Radic Biol Med. 28(7):1068–1078. 10.1016/s0891-5849(00)00196-9 10832068

[34] Muna ZA, Gudbrandsen OA, Wergedahl H, Bohov P, Skorve J, Berge RK (2002) Inhibition of rat lipoprotein oxidation after tetradecylthioacetic acid feeding. Biochem Pharmacol. 63(6):1127–1135. 10.1016/s0006-2952(01)00934-0 11931845

[35] Bjorndal B, Grimstad T, Cacabelos D, Nylund K, Aasprong OG, Omdal R, et al (2013) Tetradecylthioacetic Acid Attenuates Inflammation and Has Antioxidative Potential During Experimental Colitis in Rats. Digestive Diseases and Sciences. 58(1):97–106. 10.1007/s10620-012-2321-2 22855292

[36] Vigerust NF, Cacabelos D, Burri L, Berge K, Wergedahl H, Christensen B, et al (2012) Fish oil and 3-thia fatty acid have additive effects on lipid metabolism but antagonistic effects on oxidative damage when fed to rats for 50 weeks. J Nutr Biochem. 23(11):1384–1393. 10.1016/j.jnutbio.2011.08.006 22221672

